# The association between meteorological factors and influenza incidence in Taiwan: Regional heterogeneity and subtropical climate variability

**DOI:** 10.1371/journal.pone.0355559

**Published:** 2026-08-13

**Authors:** Po-Yueh Chen, Luong Huu Dang, Shih-Han Hung

**Affiliations:** 1 Department of Otolaryngology, Wan Fang Hospital, Taipei Medical University, Taipei, Taiwan; 2 Department of Otolaryngology, School of Medicine, College of Medicine, Taipei Medical University, Taipei, Taiwan; 3 Department of Otolaryngology, School of Medicine, University of Medicine and Pharmacy at Ho Chi Minh city, Ho Chi Minh City, Vietnam; 4 Department of Otolaryngology, Mennonite Christian Hospital, Hualien, Taiwan; 5 International Master/Ph.D. Program in Medicine, College of Medicine, Taipei Medical University, Taipei, Taiwan; Stanford University School of Medicine, UNITED STATES OF AMERICA

## Abstract

The relationship between meteorological factors and influenza seasonality is well-established in temperate regions but remains complex and less understood in subtropical climates. This study investigates the association between key weather variables and influenza-like illness (ILI) incidence in Taiwan using real observational data from 2020 to 2025. We conducted a retrospective ecological study using publicly available data from the Taiwan Centers for Disease Control and the Central Weather Administration. Based on 4,642 complete county-week observations across 17 counties/cities, we examined four meteorological factors: temperature, relative humidity, atmospheric pressure, and precipitation. We found that relative humidity showed the strongest negative correlation with ILI incidence (r = −0.147, p < 0.001; Spearman ρ = −0.196, p < 0.001), followed by atmospheric pressure (r = −0.096, p < 0.001). Temperature and precipitation did not show statistically significant Pearson correlations (r = +0.007, p = 0.625; r = +0.020, p = 0.175, respectively). Stratified analysis across five geographic regions revealed marked heterogeneity: relative humidity was most strongly negatively correlated with ILI in Northern Taiwan (r = −0.223, p < 0.001, n = 1,095) and Central Taiwan (r = −0.196, p < 0.001, n = 934), weakly negatively associated in Southern Taiwan (r = −0.072, p = 0.032, n = 898), and paradoxically positively correlated in Eastern Taiwan (r = +0.089, p = 0.012, n = 790) and the Outlying Islands (r = +0.144, p < 0.001, n = 925). The multiple regression model including all four meteorological variables yielded R^2^ = 0.045, indicating that these factors collectively explain only 4.5% of the variance in ILI incidence. In Taiwan's subtropical climate, relative humidity is the most consistent meteorological predictor of influenza activity, yet its effect is geographically heterogeneous and even reverses direction in eastern and island regions. These findings underscore the need for region-specific analysis when investigating meteorological drivers of influenza in subtropical settings.

## Introduction

Influenza, a highly contagious respiratory illness, poses a significant global public health burden, causing an estimated 290,000–650,000 deaths annually [1]. The seasonality of influenza is a well-documented phenomenon, particularly in temperate climates where epidemics typically occur during cold, dry winter months [2]. This predictable pattern is strongly linked to meteorological factors, with low temperatures and low absolute humidity being key drivers of virus survival and transmission [3,4].

However, in tropical and subtropical regions, the seasonality of influenza is far more complex and less consistent. Some regions experience semi-annual peaks, while others have year-round circulation with less defined seasonality [5,6]. This variability suggests that the relationship between weather and influenza is not universal and may be influenced by a different set of environmental and social factors. Taiwan, with its subtropical climate [7], dense population, and high-quality public health surveillance system, provides an ideal setting to investigate these complex interactions.

Previous research has yielded conflicting results. While some studies in subtropical areas have linked influenza activity to cool, dry conditions similar to temperate zones [8], others have associated it with rainy seasons and high humidity [9,10]. This discrepancy highlights the need for more localized, region-specific research. A critical challenge in large-scale ecological studies is the potential for geographic heterogeneity, where the strength or direction of an association may differ substantially between subregions, and aggregated analyses may obscure these important differences [11].

This study aims to investigate the association between four key meteorological factors (temperature, relative humidity, atmospheric pressure, and precipitation) and influenza-like illness (ILI) incidence across Taiwan from 2020 to 2025. By using real, publicly available observational data from the Taiwan CDC and the Central Weather Administration, we seek to identify the most influential weather predictors of influenza activity and to examine whether these associations differ by geographic region and season. Wind speed was excluded from this analysis due to the potential confounding influence of typhoon-related extreme wind events, which are not representative of routine meteorological conditions and could bias the correlation estimates.

## Methods

### Study design and data sources

We conducted a retrospective ecological study using publicly available, aggregated data. No individual patient information was used, and the study was exempt from institutional review board (IRB) review.

Influenza Data: Weekly ILI case counts from 2020 to 2025 were obtained from the Taiwan Centers for Disease Control (CDC) open data platform (https://data.cdc.gov.tw/dataset/rods-influenza). This dataset includes weekly case numbers stratified by county and age group.

Meteorological Data: Real observational weather data were sourced from the Central Weather Administration (CWA) CODiS (Climate Data Service) system (https://opendata.cwa.gov.tw/). We downloaded weekly summary data from weather stations across Taiwan for the period of 2020–2025. The dataset included four meteorological variables: mean temperature (°C), relative humidity (%), atmospheric pressure (hPa), and precipitation (mm). Wind speed was excluded from the analysis because typhoon events introduce extreme outlier values that are not representative of routine climate conditions and could distort correlation estimates. Each weather station was assigned to the corresponding county/city administrative unit, and weekly averages were computed for each county.

### Data processing and integration

Weather Data Processing: The downloaded CODiS data, provided in a fixed-width text format, was parsed to extract monthly values for each of the 64 stations. Each station was mapped to its corresponding county. For counties with multiple stations, the monthly meteorological values were averaged.

Temporal Alignment: To align the monthly weather data with the weekly ILI data, the monthly weather values were assigned to each week falling within that month. For precipitation, the monthly total was divided by 4.33 to estimate a weekly average.

Data Integration: The weekly ILI data and the processed weekly weather data were merged into a single dataset based on county and year/week.

Data Cleaning: The integrated dataset was cleaned by removing records with missing values for key variables. Outlier data points (e.g., temperature below 0°C or above 40°C) were also excluded. The final analysis was performed on a dataset of 4,642 county-week observations (17 counties/cities, 2020–2025). Five counties (Taoyuan City, Hsinchu County, Changhua County, Yunlin County, and Kaohsiung City) were excluded from the primary analysis due to incomplete or unverifiable meteorological station-to-county mapping in the CODiS dataset for the study period. The representativeness of the included 17 counties was verified by confirming that each county had a dedicated weather station with continuous records throughout the study period. Sensitivity analyses including all 22 counties are reported in the Supplementary Materials.

### Statistical analysis

All statistical analyses were performed using Python with the pandas, scipy, and scikit-learn libraries. Descriptive Statistics: We calculated means, standard deviations, and ranges for all meteorological variables and for ILI incidence. Correlation Analysis: Pearson correlation coefficients (r) were calculated to assess the linear association between each meteorological variable and ILI incidence. We performed this analysis on the entire dataset, and also stratified by season (Winter: Dec–Feb, Spring: Mar–May, Summer: Jun–Aug, Fall: Sep–Nov) and geographic region. For regional stratification, the 17 counties/cities were grouped into five regions based on geographic location and climatic characteristics: Northern Taiwan (Taipei City, New Taipei City, Keelung City, Hsinchu City; n = 1,095 county-weeks), Central Taiwan (Taichung City, Miaoli County, Nantou County; n = 934), Southern Taiwan (Tainan City, Chiayi City, Chiayi County, Pingtung County; n = 898), Eastern Taiwan (Yilan County, Hualien County, Taitung County; n = 790), and Outlying Islands (Penghu County, Kinmen County, Lienchiang County; n = 925). Spearman’s rank correlation (ρ) was also computed to assess robustness to non-linear associations.

Regression Analysis: A multiple linear regression model was fitted to quantify the collective effect of all four meteorological variables on ILI incidence. The coefficient of determination (R^2^) was used to measure the proportion of variance in ILI incidence explained by the model.

### Ethics statement

This study was conducted in accordance with the ethical principles of the Declaration of Helsinki. As this study utilized only de-identified, publicly available surveillance data obtained from the Taiwan Centers for Disease Control (Taiwan CDC) and the Central Weather Administration, no individual patient data were collected or accessed. The study was reviewed and granted an exemption from full ethical review by the Institutional Review Board/Ethics Committee of Mennonite Christian Hospital, Hualien, Taiwan (IRB approval number: 26-01-005). Informed consent was waived by the Institutional Review Board/Ethics Committee of Mennonite Christian Hospital, as the study involved the analysis of pre-existing, anonymized aggregate data with no direct involvement of human subjects.

## Results

**Fig 1:**
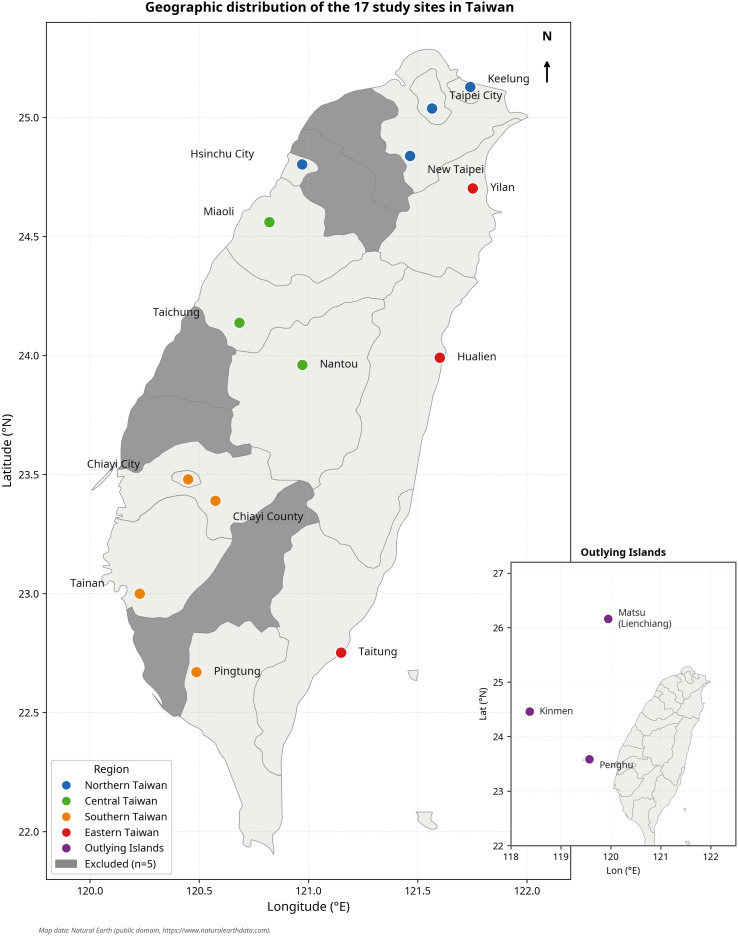
Study area and geographic distribution of the 17 counties/cities included in the analysis, grouped into five regions. Northern Taiwan: Taipei City, New Taipei City, Keelung City, and Hsinchu City (blue). Central Taiwan: Taichung City, Miaoli County, and Nantou County (green). Southern Taiwan: Tainan City, Chiayi City, Chiayi County, and Pingtung County (orange). Eastern Taiwan: Yilan County, Hualien County, and Taitung County (red). Outlying Islands: Penghu County, Kinmen County, and Lienchiang County (purple). Five counties/cities (Taoyuan City, Hsinchu County, Changhua County, Yunlin County, and Kaohsiung City) were excluded due to incomplete meteorological station-to-county mapping. Map data from Natural Earth (https://www.naturalearthdata.com), released under public domain.

The weekly ILI incidence in Taiwan from 2020 to 2025 showed distinct temporal patterns (Fig 1). The data revealed notable suppression during the COVID-19 pandemic period (2020–2022), followed by a resurgence in subsequent years.

### Overall correlation

**Fig 2:**
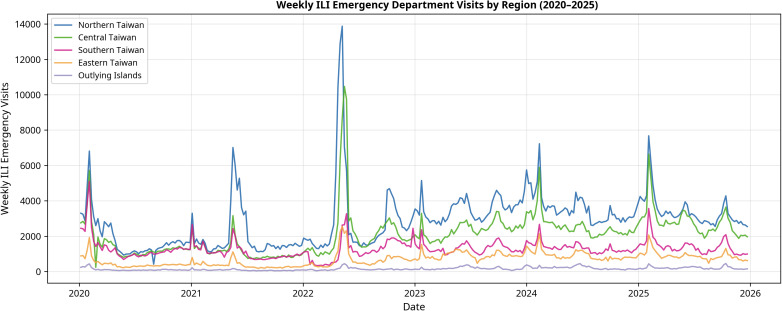
Time series of weekly influenza-like illness (ILI) emergency department visit counts in Taiwan from January 2020 to December 2025, stratified by the five geographic regions (Northern, Central, Southern, Eastern Taiwan, and Outlying Islands). The data demonstrate seasonal fluctuations with notable suppression during the COVID-19 pandemic period (2020–2022) due to non-pharmaceutical interventions, followed by a resurgence in 2023–2025. Regional differences in the magnitude and timing of ILI peaks are apparent, with Northern and Central Taiwan showing more pronounced winter peaks. Data source: Taiwan Centers for Disease Control open data platform.

Overall, we observed statistically significant correlations between ILI incidence and two of the four meteorological factors examined (Fig 2). Relative humidity showed the strongest negative correlation (r = −0.147, p < 0.001), followed by atmospheric pressure (r = −0.096, p < 0.001). Temperature (r = +0.007, p = 0.625) and precipitation (r = +0.020, p = 0.175) did not reach statistical significance. To assess the robustness of these findings and account for potential non-linear relationships, we also computed Spearman’s rank correlation. The Spearman’s ρ for relative humidity was −0.196 (p < 0.001), consistent with the Pearson correlation and confirming the robustness of the negative association between humidity and ILI incidence.

### Regional analysis and regional heterogeneity

**Fig 3:**
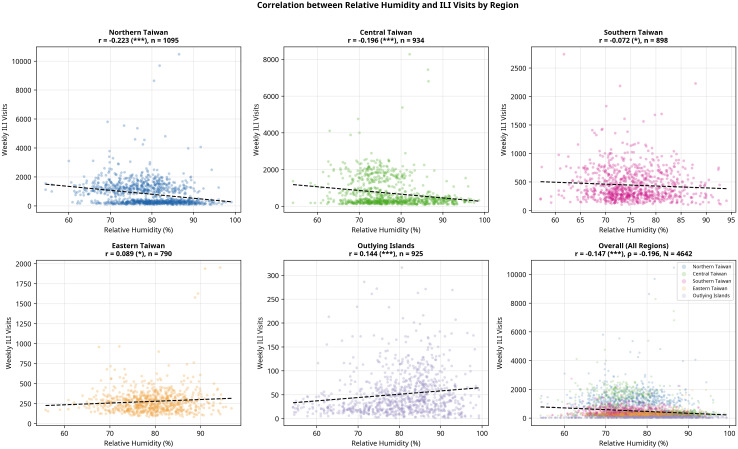
Scatter plots showing the relationship between relative humidity (%) and weekly ILI incidence across all 4,642 county-week observations (Overall panel) and stratified by five geographic regions. Each point represents a weekly observation from a specific county. Pearson correlation coefficients (r) and p-values are displayed for each panel. The overall panel shows a negative correlation (r = −0.147, p < 0.001). Regional stratification reveals marked heterogeneity: Northern Taiwan (r = −0.223, p < 0.001, n = 1,095) and Central Taiwan (r = −0.196, p < 0.001, n = 934) show the strongest negative associations, Southern Taiwan shows a weak negative association (r = −0.072, p = 0.032, n = 898), while Eastern Taiwan (r = +0.089, p = 0.012, n = 790) and Outlying Islands (r = +0.144, p < 0.001, n = 925) exhibit positive correlations.

**Fig 4:**
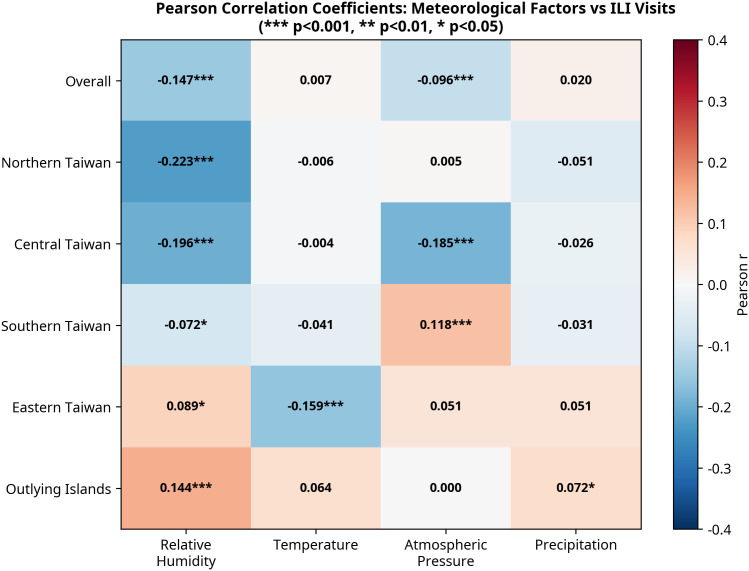
Heatmap of Pearson correlation coefficients between four meteorological factors (relative humidity, temperature, atmospheric pressure, and precipitation) and weekly ILI incidence, stratified by overall and five geographic regions. Color intensity indicates the magnitude of the correlation (blue: negative; red: positive). Statistical significance is indicated by asterisks (*** p < 0.001, ** p < 0.01, * p < 0.05). The heatmap reveals that relative humidity is the most consistent predictor across regions, with direction and magnitude varying substantially by region.

**Fig 5:**
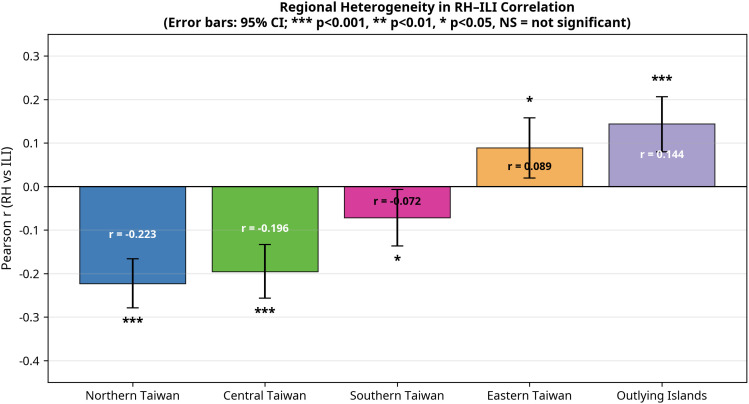
Regional heterogeneity in the association between relative humidity and ILI incidence across five geographic regions of Taiwan. Bar chart displaying Pearson correlation coefficients (r) for the humidity–ILI relationship in each region (Northern, Central, Southern, Eastern Taiwan, and Outlying Islands), alongside the overall aggregated correlation. Error bars represent 95% confidence intervals. Bars are color-coded by direction (blue: negative correlation; red: positive correlation). The figure demonstrates that the overall negative correlation (r = −0.147) masks substantial regional heterogeneity, including a reversal of direction in Eastern Taiwan (r = +0.089) and the Outlying Islands (r = +0.144), underscoring the critical importance of region-specific analysis in subtropical epidemiological research.

Stratifying the analysis by geographic region revealed striking heterogeneity in the humidity–ILI relationship across all five regions (Fig 3, Fig 4). In Northern Taiwan, relative humidity was strongly negatively correlated with ILI incidence (r = −0.223, p < 0.001, n = 1,095), indicating that higher humidity is associated with lower influenza activity. Central Taiwan showed a similarly strong negative association (r = −0.196, p < 0.001, n = 934). In Southern Taiwan, the negative association was markedly attenuated (r = −0.072, p = 0.032, n = 898). Most notably, Eastern Taiwan exhibited a statistically significant positive correlation (r = +0.089, p = 0.012, n = 790), and the Outlying Islands showed the strongest positive association (r = +0.144, p < 0.001, n = 925), indicating that higher humidity is associated with higher influenza activity in these regions. This marked regional heterogeneity, including a reversal of direction in the eastern and island regions, demonstrates that the overall negative correlation (r = −0.147) is driven predominantly by the northern and central regions, while eastern Taiwan and the outlying islands exhibit distinct epidemiological patterns (Fig 5).

### Regression analysis

The multiple linear regression model including all four meteorological variables (temperature, relative humidity, atmospheric pressure, and precipitation) was statistically significant (F-statistic p < 0.001) but had limited explanatory power, with a coefficient of determination (R^2^) of 0.045. This indicates that these four weather variables, when considered together, explain only 4.5% of the variance in ILI incidence, suggesting that meteorological factors alone are insufficient to fully explain influenza transmission dynamics in Taiwan.

## Discussion

This study, using real observational data from Taiwan, reveals that the relationship between meteorological factors and influenza is weak and highly context-dependent, a finding that contrasts with studies from temperate regions but aligns with the known complexity of influenza dynamics in the subtropics [5,6,12]. The most striking finding is the marked regional heterogeneity in the humidity–ILI relationship across Taiwan’s five geographic regions, including a reversal of direction in Eastern Taiwan and the Outlying Islands.

The regional heterogeneity observed in this study is both quantitatively and directionally striking. While the aggregated data show a weak negative correlation (r = −0.147), stratifying by region reveals a pronounced negative correlation in Northern Taiwan (r = −0.223, p < 0.001) and Central Taiwan (r = −0.196, p < 0.001), a weak negative association in Southern Taiwan (r = −0.072, p = 0.032), and a reversal to positive correlations in Eastern Taiwan (r = +0.089, p = 0.012) and the Outlying Islands (r = +0.144, p < 0.001). This divergence illustrates how aggregated ecological analyses can obscure important geographic variation in the meteorological determinants of influenza transmission [11,13]. The pattern observed here represents a true directional reversal in the eastern and island regions, underscoring the critical importance of stratified, region-specific analysis in epidemiological research.

This regional divergence is not random and is likely rooted in both climatic and socio-demographic differences across Taiwan’s diverse geography. Northern and Central Taiwan have denser urban populations, more continental climate influences, and lower baseline humidity in winter—conditions that facilitate the classic humidity–influenza relationship observed in temperate zones [3,4]. In contrast, Eastern Taiwan and the Outlying Islands experience a more maritime climate with consistently high humidity year-round, lower population density, and limited healthcare access. In these settings, the positive humidity–ILI correlation may reflect confounding by seasonal healthcare-seeking behavior: during rainy, high-humidity seasons, residents may be more likely to visit emergency departments for any respiratory illness, inflating ILI counts independent of true influenza transmission. Additionally, the small populations of these regions (particularly Kinmen, Lienchiang, and Penghu) mean that a small number of outbreak events can disproportionately influence the correlation coefficient. The sparse population and limited medical resources may also result in delayed care-seeking, with patients presenting only when symptoms are severe, further distorting the temporal relationship between weather and ILI visits [14,15].

Recent laboratory studies suggest that influenza virus stability in aerosols follows a U-shaped curve with respect to relative humidity, with optimal survival at both very low (<20%) and very high (>80%) humidity levels [16,17]. This mechanistic complexity may partly explain why the humidity–ILI relationship is not uniformly negative across all regions of Taiwan. In the Outlying Islands, where relative humidity frequently exceeds 80%, the high-humidity survival advantage of the virus may contribute to the observed positive correlation. However, it is important to note that the ecological nature of this study precludes causal inference, and the positive correlations in eastern and island regions likely reflect a complex interplay of virological, behavioral, and healthcare access factors.

Furthermore, the study by Lei et al. (2023) in China found that indoor RH, not outdoor RH, was the primary driver of influenza seasonality in subtropical climates [18]. Our study uses outdoor meteorological data, which may not accurately reflect indoor conditions where transmission actually occurs. The discrepancy between indoor and outdoor humidity is likely greater in urban areas with air conditioning (Northern and Central Taiwan) than in rural Eastern Taiwan and the Outlying Islands, where natural ventilation is more common. This differential indoor-outdoor humidity relationship may contribute to the regional heterogeneity we observed. Recent modeling studies have further demonstrated that the interplay between climate variables and influenza dynamics is highly region-specific, with temperature and humidity effects modulated by local population density, mobility, and immunity levels [19–22].

This finding has profound implications for public health practice in Taiwan. It suggests that a one-size-fits-all public health message regarding humidity and influenza risk is inappropriate for Taiwan’s diverse geographic and climatic landscape. Public health authorities should consider region-specific surveillance thresholds and communication strategies. For Northern and Central Taiwan, where the negative humidity–ILI relationship is strongest, low-humidity winter periods may serve as a useful trigger for enhanced influenza surveillance and vaccination campaigns. For Eastern Taiwan and the Outlying Islands, meteorological factors appear to be poor predictors of influenza risk, and surveillance systems should rely more heavily on virological data and healthcare utilization patterns rather than weather-based early warning systems.

The very low R^2^ value (0.045) from our regression model strongly suggests that meteorological factors are not the primary drivers of influenza transmission in Taiwan. Non-meteorological factors such as population immunity, travel patterns, school holidays, and public health interventions (e.g., mask-wearing, vaccination campaigns) play a much larger role [14,15]. The COVID-19 pandemic (2020–2023) and the associated non-pharmaceutical interventions likely acted as a major confounder during our study period, further diminishing the relative influence of weather [23].

This study has several limitations. First, the use of monthly average weather data to represent weekly conditions is a source of ecological fallacy, as short-term weather fluctuations are not captured. Second, the study did not account for other critical variables like vaccination rates, school schedules, or population mobility. Third, five counties (Taoyuan City, Hsinchu County, Changhua County, Yunlin County, and Kaohsiung City) were excluded from the primary analysis due to incomplete meteorological station-to-county mapping, which may limit the generalizability of findings to these areas. Fourth, the small sample sizes in Eastern Taiwan and the Outlying Islands (particularly the three island counties with a combined n = 925) mean that the positive correlations observed in these regions should be interpreted with caution. Finally, using ILI data as a proxy for confirmed influenza cases may introduce misclassification bias, as ILI can be caused by other respiratory pathogens.

In conclusion, this study demonstrates that in Taiwan’s subtropical climate, meteorological factors have a weak and highly region-dependent association with influenza incidence. Relative humidity emerged as the most consistent predictor overall (r = −0.147, p < 0.001), with the strongest negative effect in Northern Taiwan (r = −0.223) and Central Taiwan (r = −0.196), a weak negative effect in Southern Taiwan (r = −0.072), and paradoxical positive correlations in Eastern Taiwan (r = +0.089) and the Outlying Islands (r = +0.144). The limited explanatory power of the four-factor regression model (R^2^ = 0.045) underscores that meteorological variables alone are insufficient to explain influenza dynamics in subtropical settings. The marked regional heterogeneity observed in this study highlights the critical importance of stratified, region-specific analysis in epidemiological research, and cautions against over-generalizing findings from aggregated national-level analyses. Future studies should incorporate additional drivers such as population immunity, vaccination coverage, behavioral factors, and indoor climate data to develop more comprehensive predictive models for influenza surveillance in subtropical regions.
